# Strain of Multidrug-Resistant *Salmonella* Newport Remains Linked to Travel to Mexico and U.S. Beef Products — United States, 2021–2022

**DOI:** 10.15585/mmwr.mm7245a3

**Published:** 2023-11-10

**Authors:** Laura Ford, Zachary Ellison, Colin Schwensohn, Isabel Griffin, Meseret G. Birhane, Andrea Cote, Gamola Z. Fortenberry, Selam Tecle, Jeffrey Higa, Samantha Spencer, Brianna Patton, Jaimini Patel, Julie Dow, Azarnoush Maroufi, Amy Robbins, Danielle Donovan, Conor Fitzgerald, Sierra Burrell, Beth Tolar, Jason P. Folster, Laura A. Cooley, Louise K. Francois Watkins

**Affiliations:** ^1^Division of Foodborne, Waterborne, and Environmental Diseases, National Center for Emerging and Zoonotic Infectious Diseases, CDC; ^2^ASRT, Inc., Smyrna, Georgia; ^3^Epidemic Intelligence Service, CDC; ^4^Food Safety and Inspection Service, U.S. Department of Agriculture, Washington, DC; ^5^California Department of Public Health; ^6^Texas Department of State Health Services; ^7^Pueblo Department of Public Health and Environment, Pueblo, Colorado; ^8^Los Angeles County Department of Public Health, Los Angeles, California; ^9^Illinois Department of Public Health; ^10^Health & Human Services Agency, San Diego, California; ^11^New York State Department of Health; ^12^Michigan Department of Health & Human Services; ^13^Arizona Department of Health Services; ^14^Animal and Plant Health Inspection Service, U.S. Department of Agriculture, Washington, DC.

**Summary**

**What is already known about this topic?**

CDC monitors illness from a persisting, multidrug-resistant strain of *Salmonella* Newport linked to travel to Mexico, beef products obtained in the United States, and cheese obtained in Mexico.

**What is added by this report?**

The number of human infections with this strain doubled in 2021 from the 3-year baseline. Travel to Mexico remained a risk factor. Two multistate outbreaks were investigated; beef products obtained in the United States were the suspected vehicle in one outbreak and the confirmed vehicle in the other.

**What are the implications for public health practice?**

Safe food and drink consumption practices while traveling and at home, and interventions along the food production chain to ensure beef product safety might help prevent illness.

## Abstract

In 2016, CDC identified a multidrug-resistant (MDR) strain of *Salmonella enterica* serotype Newport that is now monitored as a persisting strain (REPJJP01). Isolates have been obtained from U.S. residents in all 50 states and the District of Columbia, linked to travel to Mexico, consumption of beef products obtained in the United States, or cheese obtained in Mexico. In 2021, the number of isolates of this strain approximately doubled compared with the 2018–2020 baseline and remained high in 2022. During January 1, 2021– December 31, 2022, a total of 1,308 isolates were obtained from patients, cattle, and sheep; 86% were MDR, most with decreased susceptibility to azithromycin. Approximately one half of patients were Hispanic or Latino; nearly one half reported travel to Mexico during the month preceding illness, and one third were hospitalized. Two multistate outbreak investigations implicated beef products obtained in the United States. This highly resistant strain might spread through travelers, animals, imported foods, domestic foods, or other sources. Isolates from domestic and imported cattle slaughtered in the United States suggests a possible source of contamination. Safe food and drink consumption practices while traveling and interventions across the food production chain to ensure beef safety are necessary in preventing illness.

## Introduction

In 2016, CDC identified a multidrug-resistant (MDR) strain of *Salmonella enterica* serotype Newport that is now monitored as a persisting strain* named REPJJP01 and includes isolates from U.S. residents in all 50 states and the District of Columbia. A 2018–2019 investigation found that persons with these infections reported traveling to Mexico or consuming beef products obtained in the United States or cheese obtained in Mexico (*1*). During 2021, CDC’s PulseNet database received an increase of REPJJP01 reports, which prompted this investigation. This report analyzed isolates identified during 2021–2022.

## Methods

### Isolate Sequencing and Determination of Antimicrobial Resistance

*Salmonella* isolates underwent whole genome sequencing at U.S. public health laboratories and federal agencies, and the resulting sequence data were uploaded to the PulseNet *Salmonella* National Database and analyzed by core genome multilocus sequence typing (cgMLST) (*2*). CDC defined REPJJP01 as a strain of *S.* Newport with a range of 0–21 allele differences by cgMLST. Isolate genomes were screened for resistance determinants and assigned predicted resistance patterns using ResFinder^†^ drug keys as part of surveillance through the National Antimicrobial Resistance Monitoring System. Isolates were defined as MDR if they contained resistance determinants for three or more antimicrobial classes.

### Data Analyses 

Public health officials obtained travel and food exposure information from interviews and medical records of patients with the REPJJP01 strain isolated; some data were not available for all patients. Characteristics of patients with illness onset dates^§^ and food and animal samples with collection dates during January 1, 2021–December 31, 2022, were summarized, and proportions were compared using chi-square tests; p<0.05 was considered statistically significant. R statistical software (version 4.2.3; R Foundation) was used to conduct these analyses. CDC investigated multistate outbreaks among nontravelers with support from local and state health departments and the U.S. Department of Agriculture’s Food Safety and Inspection Service. This activity was reviewed by CDC, deemed not research, and was conducted consistent with applicable federal law and CDC policy.^¶^

## Results

### Trends in Isolate Detection

In 2021, the total number of human isolates (641) approximately doubled from the average annual baseline number of cases detected during 2018–2020** (315) and remained high in 2022 (641) (Figure). Nonhuman isolates included 25 from cattle (beef products and cecal^††^ samples) and one from a sheep (cecal sample) from 13 U.S. states.^§§^

**FIGURE:**
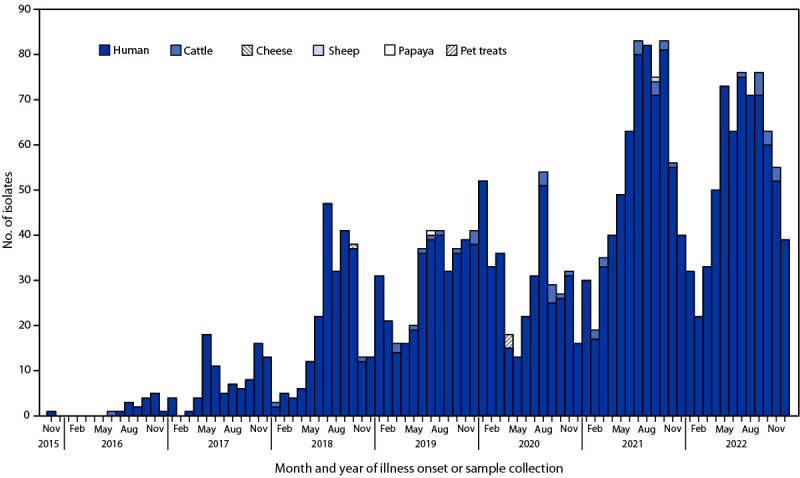
Month and year of illness onset* or sample collection^†^ and source type for isolates of multidrug resistant *Salmonella* Newport strain REPJJP01 — United States, 2016–2022 * Some illness onset dates for human cases have been estimated based on the isolation date. ^†^ Nonhuman isolates.

### Patient and Isolate Characteristics

The median patient was aged 37.5 years (IQR = 23–55 years), 52% were female, 56% were Hispanic or Latino (Hispanic), and 19% and 17% were California and Texas residents, respectively (Table). Among all patients with hospitalization data available (721), one third (247, 33%) were hospitalized, and two died (<1%). The percentage of Hispanic patients who were hospitalized (40%) was higher than that of non-Hispanic White patients (24%, p<0.001) or non-Hispanic patients of other races (22%, p = 0.03). Most patient isolates (1,141, 89%) were resistant or had decreased susceptibility to at least one antibiotic recommended for empiric or alternative treatment (*3*–*5*), and 1,110 (87%) were MDR. Most food and cecal isolates also had antimicrobial resistance; 65% of food and cecal isolates were MDR.

**TABLE:** Patient and clinical isolate characteristics of infections with multidrug-resistant *Salmonella* Newport strain REPJJP01 — United States, 2021–2022

Characteristic	Year No./Total no.* (%)		
	2021	2022	2021–2022
**Patients, total no.**	**641**	**641**	**1,282**
**Sex**			
Female	321/624 (51)	338/634 (53)	659/1,258 (52)
Male	303/624 (49)	296/634 (47)	599/1,258 (48)
**Median age, yrs (IQR)**	34 (20–52)	41 (26–58)	38 (23–55)
**Race and ethnicity^†^**			
Hispanic or Latino	228/349 (65)	195/407 (48)	423/756 (56)
White, non-Hispanic	100/349 (29)	176/407 (43)	276/756 (37)
Other or unknown race, non-Hispanic	21/349 (6)	36/407 (9)	57/756 (8)
**Hospitalized**			
Yes	123/346 (36)	124/412 (30)	247/758 (33)
No	223/346 (64)	288/412 (70)	511/758 (67)
**Travel to Mexico**			
Yes	112/287 (39)	232/434 (53)	344/721 (48)
No	175/287 (61)	202/434 (47)	377/721 (52)
**Clinical isolates**			
**Source**			
Stool	554/632 (88)	543/634 (86)	1,097/1,266 (87)
Urine	43/632 (7)	57/634 (9)	100/1,266 (8)
Blood	24/632 (4)	22/634 (3)	46/1,266 (4)
Other^§^	11/632 (2)	12/634 (2)	23/1,266 (2)
**Antimicrobial resistance^¶^**			
Ampicillin	493/640 (77)	449/641 (70)	942/1,281 (74)
Azithromycin**	562/640 (88)	536/641 (84)	1,098/1,281 (86)
Ceftriaxone	1/640 (<1)	0/641 (—)	1/1,281 (<1)
Ciprofloxacin^††^	514/640 (80)	475/641 (74)	989/1,281 (77)
Trimethoprim-sulfamethoxazole	572/640 (89)	534/641 (83)	1,106/1,281 (86)
Multidrug resistance	574/640 (90)	536/641 (84)	1,110/1,281 (87)
No resistance	46/640 (7)	94/641 (15)	140/1,281 (11)

* Denominators may not equal totals for each year due to missing data.

^†^ Persons of Hispanic or Latino (Hispanic) origin might be of any race but are categorized as Hispanic; all racial groups are non-Hispanic.

^§^ Other source sites include abscess, fluid, iliac aneurysm, rectal swab, synovial fluid, tissue, or wound.

^¶^ Resistance was determined based on the results of antimicrobial susceptibility testing when available (37); otherwise, resistance was predicted based on whole genome sequencing. Isolates were considered to be resistant to an antimicrobial if the presence of at least one resistance mechanism known to confer resistance to that antimicrobial was present in the isolate’s genome. One isolate in 2021 was not tested by antimicrobial susceptibility testing or analyzed for resistance determinants and is not included in the denominator.

** Interpretive criteria for azithromycin resistance have not been established for *Salmonella* serotypes other than serotype Typhi. Therefore, the presence of resistance determinants for azithromycin should not be used to predict clinical efficacy.

^††^ Includes isolates with interpretation of “intermediate” or isolates carrying a single quinolone resistance gene (878; 89%); a single gene might result in interpretation of “intermediate” for ciprofloxacin on antimicrobial susceptibility testing.

Travel to Mexico remained a risk factor for infection during 2021–2022. Among 721 patients with known travel history, nearly one half (344, 48%) reported traveling to Mexico in the month before illness onset. Compared with persons without a recent history of travel to Mexico, fewer travelers to Mexico were Hispanic (43% versus 64%, p<0.001) or were hospitalized (24% versus 37%, p<0.001). Reported travel to Mexico was more prevalent in 2022 (53%) than in 2021 (39%) (p<0.001). Eleven ill nontravelers reported eating foods, including queso fresco or dried beef, purchased in Mexico by family or friends.

### Outbreak Investigations

A multistate 75-patient outbreak was investigated during fall 2021; 80% of patients in that outbreak were Hispanic, and beef, including dried beef, was the suspected vehicle. A second multistate outbreak that included 22 non-Hispanic patients was investigated during fall 2022. A strain of *S.* Newport indistinguishable from the clinical isolates was isolated in a sample of leftover ground beef from a patient. However, because the beef could not be traced back definitively to a common source, no regulatory action could be taken.  Six subclusters of REPJJP01 isolates related within 0–2 allele differences during 2021–2022 contained at least one clinical and one beef isolate; however, these small clusters did not include sufficient cases to conduct an epidemiologic investigation that could implicate a product.

## Discussion

During 2021–2022, a total of 1,282 U.S. residents had culture-confirmed infections caused by REPJJP01, an MDR strain of *S. *Newport. The total number of cases of illness is likely much larger: an additional 29 cases of *Salmonella* are estimated for each culture-confirmed case (*6*). Further, the sharp increase in cases of REPJJP01 during 2021 occurred despite an overall decrease in *Salmonella* reports during the COVID-19 pandemic (*7*). Patient exposures and outbreak sources resembled those identified in the 2018–2019 investigation (*1*), including travel to Mexico, consumption of beef and cheese products purchased in Mexico, and consumption of beef products obtained in the United States.

This strain might spread to the United States through returned travelers from Mexico, cattle born or raised in Mexico and slaughtered in the United States, or beef or cheese imported from Mexico. The increase in cases during 2021–2022 appears driven partially by travelers; the 48% of patients who reported travel to Mexico is high compared with the 9% of all patients with nontyphoidal *Salmonella* reporting any international travel.^¶¶^ In September 2022, CDC posted a Level 1 Travel Health Notice to alert travelers to Mexico about the risk for infection and to provide advice for prevention.*** Travelers should practice safe eating, drinking, cooking, and food handling habits to reduce their chance of becoming ill. Among nontravelers, the high proportion of Hispanic patients and patient reports of eating beef or cheese purchased by family or friends in Mexico suggests that beef or cheese imported from Mexico might be an important source of illness.^†††^

The REPJJP01 strain might also spread within the United States through animals or beef products. The strain has been isolated in samples collected in federally regulated slaughter and processing establishments throughout the United States and import establishments on the Southern border. The isolation of this strain from a beef product collected during a 2022 multistate outbreak investigation suggests cattle infected with *Salmonella* in the United States might serve as a potential source of contamination. During outbreaks, the source of implicated beef products is difficult to identify because patients report consuming beef products both inside and outside the home, shopper history records or receipts are often not available, and traceback is complex, with challenges including incomplete retail grinding logs and multiple products combined in retail products (*8*). Steps to prevent contamination of beef products might occur across the food production chain, including at preharvest (e.g., herd and biosecurity management), slaughter (e.g., appropriate sanitary dressing procedures and application of effective antimicrobial solutions), processing, and retail (*8*). Consumers should wash hands and surfaces after preparing raw meat at home and use a thermometer to ensure appropriate cooking temperatures are reached (*9*).

Some persons infected with REPJJP01 became seriously ill: 33% of all patients, 37% of non-travelers, and 40% of Hispanic patients were hospitalized, compared with 27% of all patients with nontyphoidal *Salmonella* infections reporting hospitalization in FoodNet data during 2021–2022. The high hospitalization rate is consistent with studies indicating that patients with antimicrobial-resistant *Salmonella* infections are more likely to be hospitalized (*10*). The increase in infections from this MDR strain is concerning because it limits treatment options, has more severe outcomes, and creates opportunities for resistance genes to spread. Clinicians should be aware of the potential for multidrug resistance in recent travelers to Mexico with salmonellosis and should order susceptibility testing to guide antimicrobial selection when treatment is warranted.^§§§^

### Limitations

The findings in this report are subject to at least three limitations. First, the number of illnesses recorded by the public health system is an underestimate of the total number of illnesses. Second, not all patients were able to be interviewed, limiting the ability to collect exposure information. Finally, the proportion of patients reporting travel might be an underestimate, because some patients were only asked about travel during the 4 or 7 days before illness onset.

### Implications for Public Health Practice

CDC continues to work with local and state health departments and federal partners to investigate cases of this strain to identify sources of infection and prevent illness. Interventions along the food production chain to ensure beef safety (*8*) and prevention measures among producers, consumers, and travelers might help to reduce illness from this persisting MDR strain. Consumers should eat beef only if it is cooked thoroughly. Travelers to Mexico should follow food safety practices while abroad (https://wwwnc.cdc.gov/travel/page/food-water-safety); to reduce the chances of illness, travelers should avoid beef (including beef jerky or dried beef) and other foods that are prepared or sold by street vendors.  Further research is needed to understand the consequences of decreased susceptibility to azithromycin on clinical outcomes for patients receiving this agent.

## Footnotes

* This strain is monitored as a reoccurring, emerging, or persisting (REP) strain; “persisting” is defined as causing illness consistently during a long period. https://www.cdc.gov/ncezid/dfwed/outbreak-response/rep-strains.html

† https://github.com/tseemann/shovill; https://github.com/phac-nml/staramr;  https://github.com/StaPH-B/resistanceDetectionCDC

§ If illness onset date was unknown, it was presumptively calculated as the isolation date minus 3 days.

¶ 45 C.F.R. part 46, 21 C.F.R. part 56; 42 U.S.C. Sect. 241(d); 5 U.S.C. Sect. 552a; 44 U.S.C. Sect.3501 et seq.

** The 3-year baseline is the average number of culture-confirmed isolates during 2018–2020.

††
https://www.fsis.usda.gov/sites/default/files/media_file/2020-07/NARMS-Salmonella-Cecal-Report-2014.pdf

§§ No samples were imported during 2021–2022.

¶¶
https://wwwn.cdc.gov/foodnetfast

***
https://wwwnc.cdc.gov/travel/notices/watch/salmonella-newport-mexico

†††
https://ask.usda.gov/s/article/Products-for-personal-consumption

§§§
https://www.cdc.gov/salmonella/general/technical/newport.html
